# Role of Organic Cation Transporter 2 in Autophagy Induced by Platinum Derivatives

**DOI:** 10.3390/ijms23031090

**Published:** 2022-01-19

**Authors:** Sara Ahmed Eltayeb, Giuliano Ciarimboli, Katrin Beul, Giovana Seno Di Marco, Vivien Barz

**Affiliations:** Medicine Clinic D, Experimental Nephrology, University Hospital of Münster, 48149 Münster, Germany; Sara.Eltayeb@ukmuenster.de (S.A.E.); katrin.beul@ukmuenster.de (K.B.); giodimarco@gmail.com (G.S.D.M.); v.barz@gmx.de (V.B.)

**Keywords:** transport, platinum derivatives, toxicity, autophagy

## Abstract

The human organic cation transporter 2 (hOCT2) mediates renal and neuronal cellular cisplatin and oxaliplatin uptake, and therefore plays a significant role in the development of side effects associated with these chemotherapeutic drugs. Autophagy is induced by cisplatin and oxaliplatin treatment and is believed to promote cell survival under stressful conditions. We examined in vitro the role of hOCT2 on autophagy induced by cisplatin and oxaliplatin. We also explored the effect of autophagy on toxicities of these platinum derivatives. Our results indicate that autophagy, measured as LC3 II accumulation and reduction in p62 expression level, is induced in response to cisplatin and oxaliplatin in HEK293-hOCT2 but not in wild-type HEK293 cells. Furthermore, inhibition of autophagy is associated with higher toxicity of platinum derivatives, and starvation was found to offer protection against cisplatin-associated toxicity. In conclusion, activation of autophagy could be a potential strategy to protect against unwanted toxicities induced by treatment with platinum derivatives.

## 1. Introduction

Cisplatin ((SP-4-2)-diammindichloridoplatin(II), CDDP) and oxaliplatin ([(1R,2R)-cyclohexane-1,2-diamine](ethanedioato-O,O’)platinum(II), OX) are platinum (Pt) derivatives which play an important role in the treatment of epithelial malignancies such as lung, head, neck, ovarian, bladder and testicular cancer [1,2,3,4]. These chemotherapeutic agents are known to target DNA, forming intrastrand and interstrand cross links, which subsequently lead to DNA damage and cellular apoptosis [3]. However, only a minor part of intracellular Pt (5 to 10%) is associated with DNA, being mostly bound to RNA, proteins, and small thiol compounds [5,6,7]. One major problem associated with chemotherapeutic protocols based on Pt derivatives is the emergence of serious unwanted side effects such as nephrotoxicity, ototoxicity, and neurotoxicity [2]. Growing evidence [8,9,10] suggests that these side effects are attributed to the interaction between Pt agents and organic cation transporters (OCTs). These transporters belong to the solute carrier (SLC) 22 family and act by mediating pH- and Na^+^-independent organic cation transport across the cell membrane [2], which is driven by the electrochemical substrate gradient. The human organic cation transporter 2 (*SLC22A2*, hOCT2) is highly expressed in the basolateral membrane of renal proximal convoluted tubules, where it mediates cellular uptake of CDDP, resulting in nephrotoxicity [2]. hOCT2 is also believed to mediate OX accumulation in dorsal root ganglia, which leads to neurotoxicity [11]. Most cancer cells do not seem to express OCT2 [12], and competition with OCT2 seems not to interfere with CDDP’s anticancer efficacy [13]. Thus, pharmacological targeting of OCT2 could be a promising strategy in preventing side effects associated with cancer therapy with Pt derivatives [11].

Chemotherapeutic agents such as CDDP [14] and OX [15] are known to cause autophagy. Autophagy promotes cell survival as a response to stress factors such as starvation [16], hypoxia and hyperthermia [17]. The process of autophagy takes place also during normal cellular life, where it is responsible for the breakdown and removal of long-lived cellular proteins, dysfunctional components such as misfolded proteins, and damaged organelles [18,19]. Under stress conditions, autophagy is crucial for cell survival [14,20]. This is attributed to the fact that, during stress, autophagy generates amino acids, fatty acids and sugars that can be further utilized for energy production. Autophagy is initiated by formation of double-membrane vesicles known as autophagosomes [14,21]. Autophagosomes surround the internal content to be degraded and then fuse with lysosomes to form autolysosomes. In these vesicles, the autophagosomal content is degraded by lysosomal hydrolases [14,21,22,23]. The formation of autophagosomes is a complex process that involves several autophagy-related proteins such as Beclin 1, Vps 34, Atg 5, Atg 12 and LC3 II [14], the latter being widely used as a reliable marker for autophagosome formation [24]. It is well-known that inhibition of autophagy under stress conditions leads to apoptosis [14,25]. Interestingly, extremely harsh conditions lead to massive autophagy activation, which results in cell death, a process known as programmed cell death type II [14,26].

hOCT2 mediates cellular uptake of several Pt derivatives [2,11]; however, its role in the development of autophagy induced by these chemotherapeutic drugs has not yet been investigated. In this work, we investigated the relationship between hOCT2 and autophagy flux induced by CDDP and OX. Furthermore, since autophagy plays a vital role in cell survival under stress conditions, we examined its effect on CDDP/OX associated cytotoxicity.

## 2. Results

### 2.1. Starvation Promotes LC3 II Accumulation in HEK293 Cells

**Figure 1:**
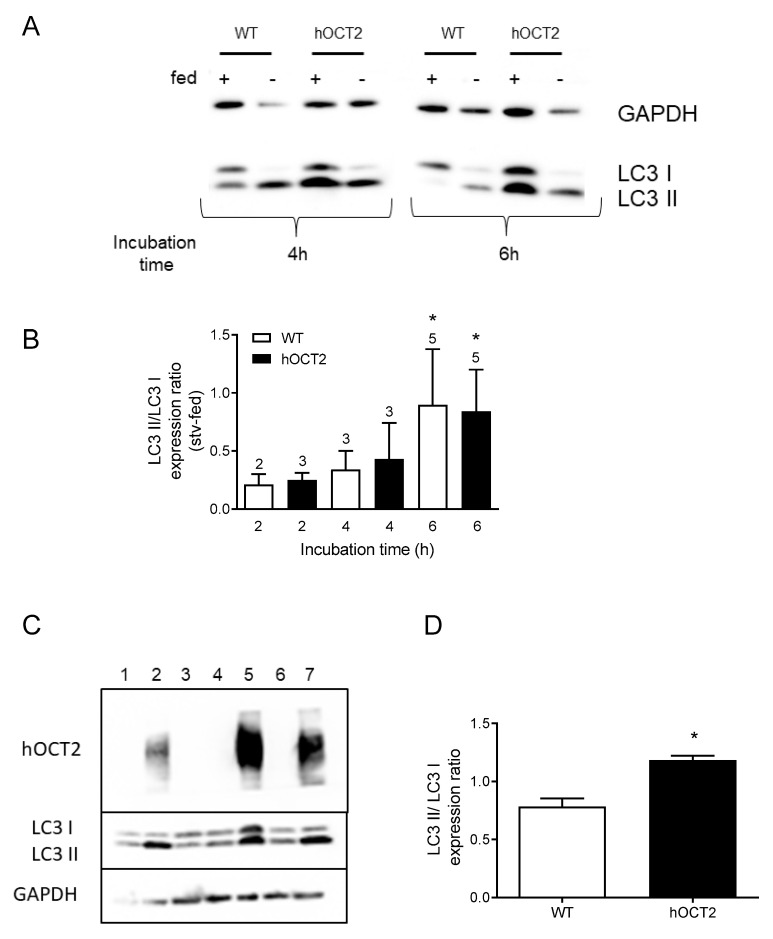
This figure shows the effects of starvation and of hOCT2 overexpression on cellular autophagy activity measured as LC3 II/I expression ratio in HEK293-WT (WT) and HEK293-hOCT2 (hOCT2) cells. Panel (**A**) shows a representative Western blot analysis of LC3 expression in cells incubated for 4 and 6 h in DMEM (fed +) or EBSS (fed −) medium, as fed or starvation condition, respectively. GAPDH was used as a loading control. Panel (**B**) shows the quantification of Western blot analyses of lysates from WT (open bars) and hOCT2 (closed bars) cells. Data (mean ± SEM) are expressed as difference between LC3 II/LC3 I ratios determined at each time point for starvation and fed conditions in WT and hOCT2 cells, respectively. The numbers above the columns show the number of independent Western blot analysis performed. * shows that there is a statistically significant autophagy activation (*p* < 0.05, comparison between starvation and fed conditions by paired *t*-test) after 6 h incubation both in WT and hOCT2 cells. Panel (**C**) shows the Western blot analysis of LC3 II/LC3 I expression ratio in WT (lanes 1, 3, 4, 6) and hOCT2 (lanes 2, 5, 7) cells with GAPDH as a loading control and hOCT2 signal as control for transporter expression. Panel (**D**) shows the quantification of the LC3 II/LC3 I expression ratio determined by Western blot analysis of lysates from WT (open column, N = 4) and hOCT2 (closed column, N = 3) cells. Data are given as mean ± SEM and * shows a statistically significant difference between WT and hOCT2 cells (*p* < 0.05, unpaired *t*-test).

The microtubule-associated protein 1A/1B-light chain 3 (LC3) is involved in several steps of the autophagy process, including autophagosome formation and autophagosome fusion with lysosomes [27]. During autophagy, LC3 I, a cytosolic form of LC3, is conjugated to phosphatidylethanolamine forming LC3 II, a modification which is widely used as an autophagy marker [24]. Indeed, autophagy activity is monitored measuring the ratio between LC3 II and LC3 I expression levels. Since starvation is a strong triggering factor of autophagy [19,28], we investigated whether LC3 I is modified to LC3 II in response to starvation. For this, HEK293-WT or -hOCT2 cells were incubated with EBSS (starvation condition) for 2, 4, or 6 h and the LC3 I and LC3 II protein expression were measured using Western blot analysis. Our results showed that 6 h starvation triggers LC3 I to LC3 II conversion in HEK293-WT and -hOCT2 cells (Figure 1A,B). Moreover, it seems that hOCT2-expressing HEK293 cells have a higher basal autophagic activity than HEK293-WT cells (Figure 1C,D).

### 2.2. Induction of Autophagy in Response to CDDP and OX Treatment

**Figure 2:**
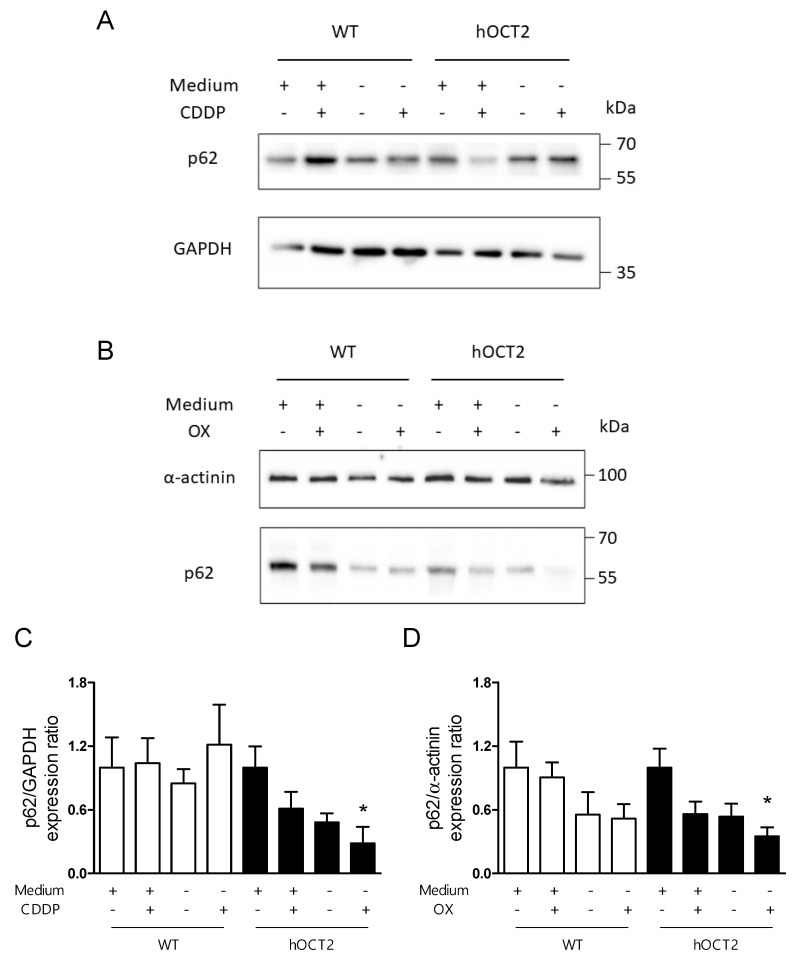
This figure shows activation of autophagy under CDDP or OX treatment of HEK293-WT (WT) and -hOCT2 (hOCT2) cells measured as p62 expression decrease. Cells were treated with CDDP (100 µM, panels (**A**,**C**)) or OX (100 µM, panels (**B**,**D**)) for 6 h under fed (medium +) or starvation (medium −) conditions, as described in the Material and Methods section. The p62 expression levels were measured using Western blot analysis and GAPDH (**A**,**C**) or *α*–actinin (**B**,**D**) were used as loading controls. Panels (**A**,**B**) show representative Western blot analysis and panels (**C**,**D**) show the quantification as mean ± SEM of 4 independent experiments (open bars = WT cells; closed bars = hOCT2 cells). * represent a statistically significant difference between the indicated group and all the others (*p* < 0.05, one way ANOVA with Tukey’s multiple comparison test). To facilitate the comparison of the effects, the expression ratio values for incubation with fed medium without CDDP or OX were set to 1 for WT and hOCT2 cells.

We next examined whether autophagy is activated in response to CDDP or OX treatment in HEK293-WT and -hOCT2 cells. In this setting, we used p62 as an autophagy marker. This protein is believed to be degraded during autophagy, and therefore reduction in p62 level reflects autophagy activation [29]. Our data showed that both CDDP and OX treatment caused a decrease in p62 expression, mainly in hOCT2-expressing HEK293 cells (Figure 2). Confirming the above-described results (Figure 1), starvation induced autophagic activity both in HEK293-WT and -hOCT2 cells. Still, only in hOCT2-expressing cells, CDDP and OX treatment further increased autophagy to statistically significant values (Figure 2C,D).

**Figure 3:**
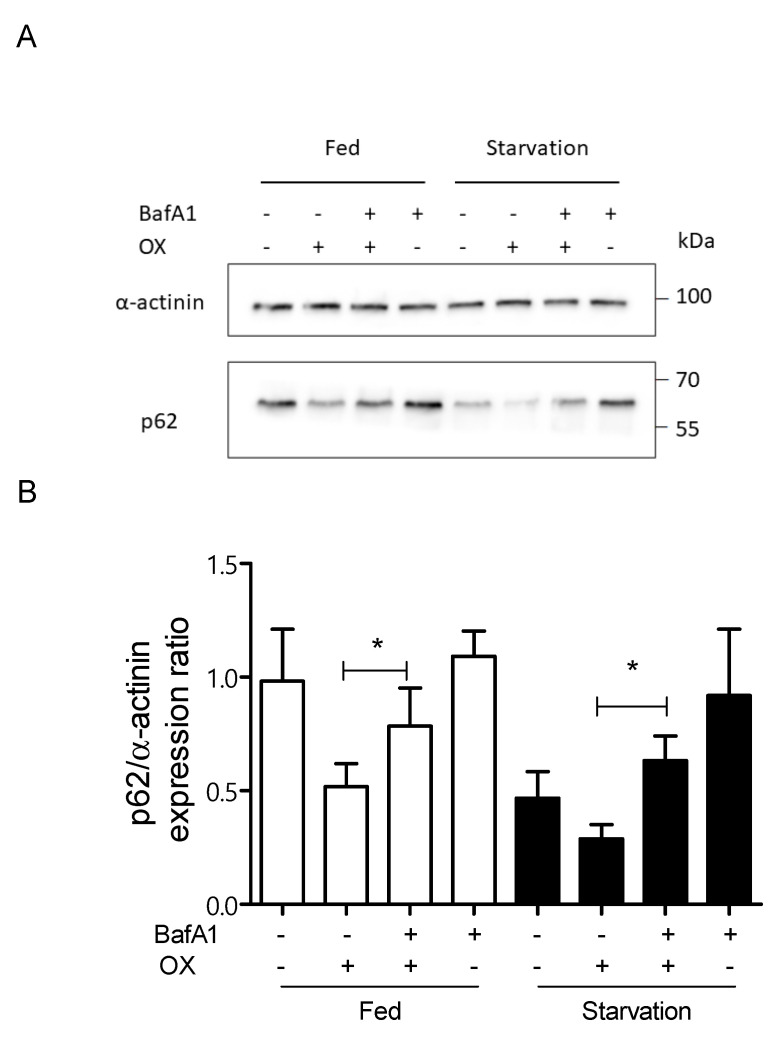
This figure shows the results of experiments aimed to confirm induction of autophagy flux by OX treatment in HEK293-hOCT2 cells using bafilomycin A1 (BafA1) as inhibitor of autophagy flux, and using p62 as autophagy marker. HEK293-hOCT2 cells were treated with OX (100 µM) for 6 h in the presence or absence of BafA1 (100 nM) under fed (open columns) or starvation (closed columns) conditions. The p62 expression levels were measured using Western blot analysis with *α*–actinin as a loading control. The panel (**A**) shows a representative Western blot analysis and panel (**B**) the quantification of 4 independent experiments. Data are expressed as mean ± SEM. * represent a statistically significant difference (*p* < 0.05, paired *t* test) between the indicated groups.

Because CDDP and OX treatment showed similar effects on p62/housekeeping protein expression ratios, focusing only on OX treatment, we investigated whether inhibition of autophagic flux with bafilomycin A1 [30] influences p62 expression. As shown in Figure 3A,B, the p62 expression level increased in cells treated with bafilomycin A1 and OX compared with cells treated only with OX.

**Figure 4:**
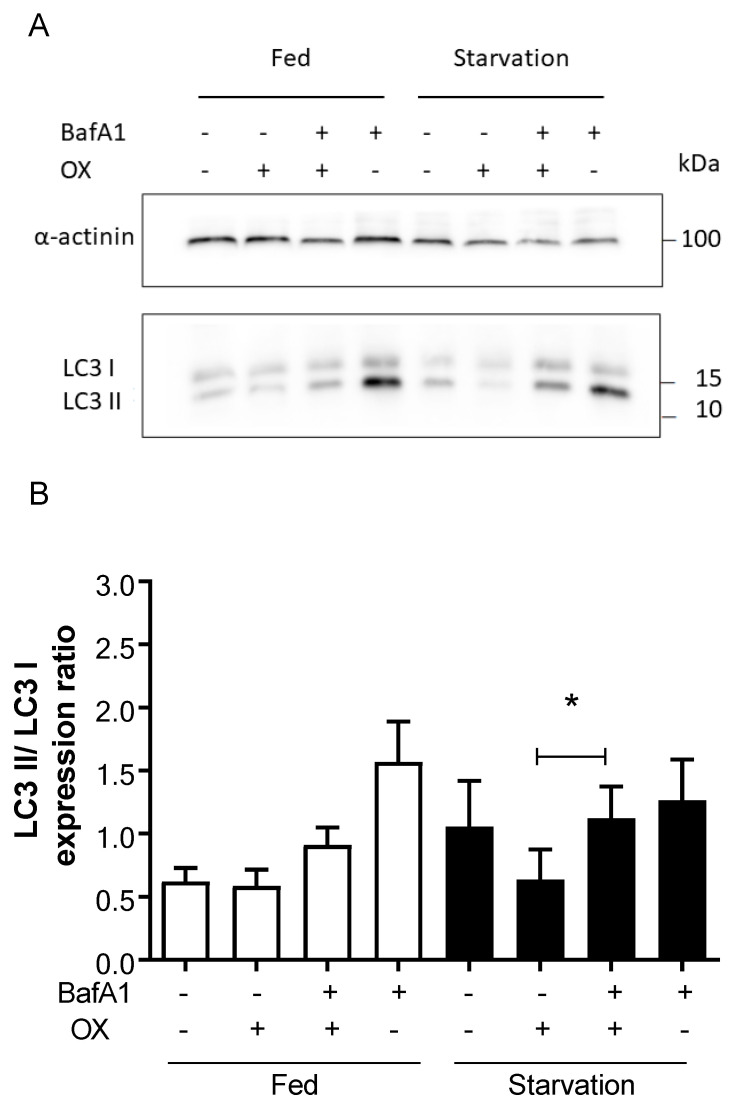
This figure shows the results of experiments aimed to confirm induction of autophagy by OX treatment in HEK293-hOCT2 cells using bafilomycin A1 (BafA1) as an inhibitor of autophagy flux and LC3 II/LC3 I expression ratio as an autophagy marker. HEK293-hOCT2 cells were treated with OX (100 µM) for 6 h in the presence or absence of bafilomycin A1 (BafA1, 100 nM) under fed or starvation conditions. The ratios between LC3 II and LC3 I expression levels were measured using Western blot analysis. *α*-actinin was used as a loading control. Panel (**A**) shows a representative Western blot analysis, and panel (**B**) shows the quantification (mean ± SEM) of 5 independent experiments. * represents a statistically significant difference between the indicated groups (*p* < 0.05, paired *t* test).

As outlined above, LC3 II accumulation serves as a reliable marker of autophagy [24]. During starvation, LC3 II itself is markedly degraded [31], however, when treating the cells with lysosomal protease inhibitors such as bafilomycin A1, this degradation is blocked and LC3 II further increases. Figure 4 shows that during starvation, LC3 II significantly accumulates in cells treated with OX and bafilomycin compared to cells treated with OX alone.

Taken together, these results not only show that bafilomycin A1 can effectively block activation of autophagy by OX, but also confirm that OX is indeed increasing autophagy in HEK293-hOCT2 cells.

Interestingly, OX incubation of HEK293-hOCT2 caused a reduction in hOCT2 protein expression, probably because autophagic degradation of OX-bound transporter (Supplementary Figure S2). Indeed, this effect was not detected under activation of autophagy by starvation.

### 2.3. MTORC1 Is Downregulated during Starvation in HEK293-WT and HEK293-hOCT2 Cells

**Figure 5:**
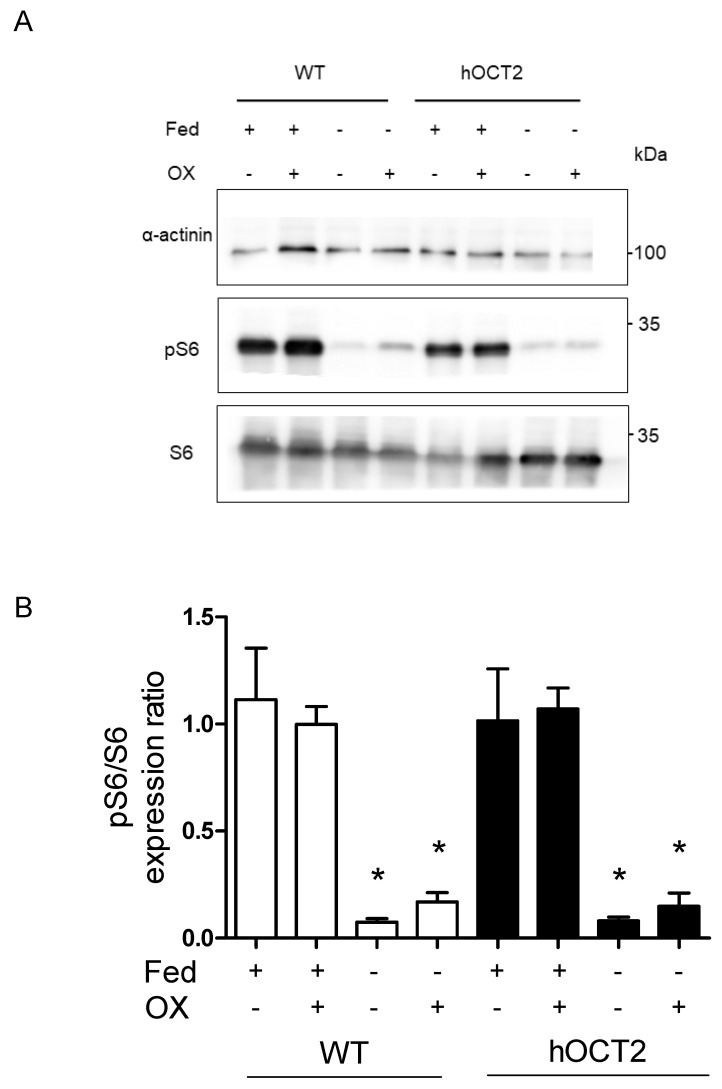
This figure shows the results of experiments aimed to measure the effect of OX incubation and/or starvation on the mTORC signaling pathway in HEK293-WT (WT) and -hOCT2 (hOCT2) cells. Activation of mTORC signaling pathway was measured by Western blot analysis of pS6/S6 expression ratio in WT (open bars) and hOCT2 (closed bars) cells under fed and starvation conditions and OX treatment. *α*-actinin served as a loading control. Panel (**A**) shows a representative Western blot analysis, panel (**B**) shows the quantification of the results (mean ± SEM) from 3 independent experiments. The mTORC signaling pathway is downregulated by starvation in both WT and hOCT2 cells. * indicate a statistically significant difference between fed and starvation conditions (*p* < 0.05, Anova with Tukey post hoc test). Treatment with 100 µM OX for 6 h under fed or starvation condition did not significantly change the activity of the mTORC pathway compared with experiments performed without OX treatment.

The mammalian target of rapamycin complex 1 (mTORC1) is a strong suppressor of autophagy. mTORC1 activation leads to phosphorylation of p70 S6-kinase, which phosphorylates its downstream protein S6 [32,33]. In this setting, we tested whether autophagy induced by OX and/or starvation treatment influences mTORC1 signaling. Reduction in pS6/S6 expression level was observed in both HEK293-WT and -hOCT2 cells during starvation, suggesting autophagy activation (Figure 5). Incubation with OX did not change pS6/S6 expression ratio (Figure 5). The same was observed when treating the cells with CDDP instead of OX (not shown).

### 2.4. Autophagy Inhibition Exacerbates CDDP- and OX-Induced Cytotoxicity

**Figure 6:**
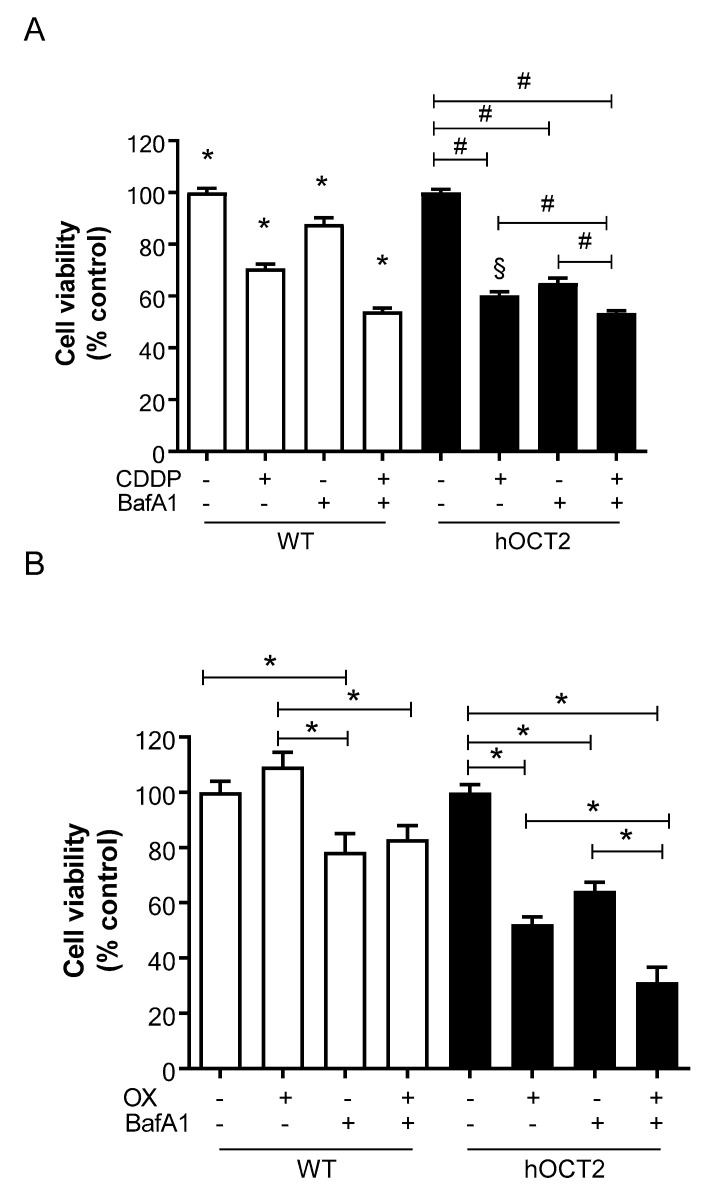
This figure shows the cytotoxic effects measured with an MTT assay in HEK293-WT (WT, open bars) and -hOCT2 (hOCT2, closed bars) cells after 6 h incubation with 100 µM CDDP (panel **A**) or 100 µM OX (panel **B**) in the presence or absence of 100 nM bafilomycin A1, followed by a 48 h postincubation with drug-free medium. Each bar shows the mean ± SEM of 6 replicates measured in three independent experiments. Panel (**A**): * represent a statistically significant difference between the indicated groups (*p* < 0.05, ANOVA with Tukey post hoc test) and all the others. # shows a statistically significant difference in the indicated groups (*p* < 0.05, ANOVA with Tukey post hoc test). § shows a statistically significant difference between the effect of treatment in WT and hOCT2 cells (*p* < 0.05, unpaired *t*-test). Panel (**B**): * show a statistically significant difference between the effect in the indicated groups (*p* < 0.05, ANOVA with Tukey post hoc test).

We also determined whether autophagy inhibition changes CDDP- and OX-associated cellular death. To do this, we incubated HEK293-WT and HEK293-hOCT2 cells with 100 µM CDDP or OX in the presence or absence of 100 nM bafilomycin A1 for 6 h. After this, the medium was removed and replaced with drug-free medium, and cells were further incubated for 48 h. At the end of this postincubation period, cell viability was determined using the MTT test. Incubation with CDDP or OX markedly induced cellular toxicity (Figure 6). Interestingly, this toxicity was more evident in HEK293-hOCT2 cells, confirming that hOCT2 plays a crucial role in the cytotoxicity of platinum derivatives [34]. Furthermore, autophagy inhibition with bafilomycin A1 enhanced CDDP- and OX-associated cytotoxicity (Figure 6).

**Figure 7:**
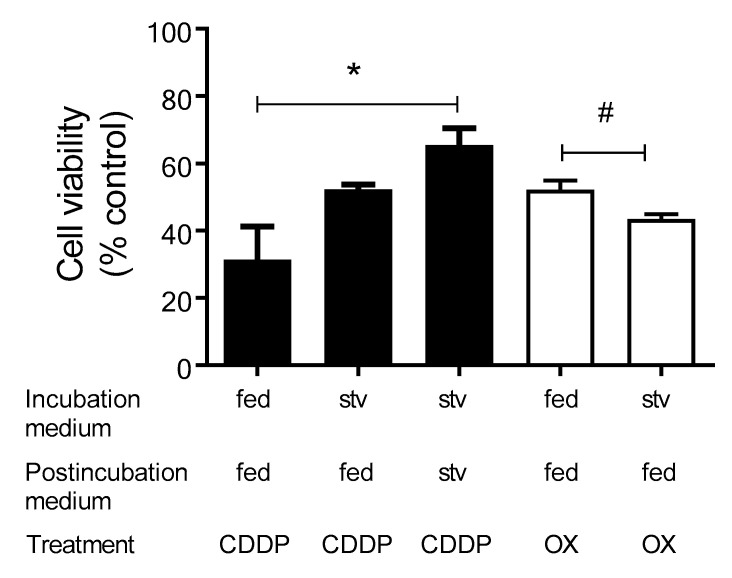
This figure shows the cytotoxic effects of 6 h incubation of HEK293-hOCT2 cells with 100 µM CDDP or OX under fed or starvation (stv) conditions followed by postincubation with DMEM (fed) or EBSS (stv, only for CDDP) for 48 h. Cell viability was measured using an MTT assay. Each bar shows the mean ± SEM of 6 replicates measured in three independent experiments. * and # show a statistically significant difference between the tested conditions (*p* < 0.05, *ANOVA with Tukey post hoc test and # unpaired *t*-test, respectively).

Accordingly, starvation—probably by triggering autophagy [19]—may protect against CDDP- and OX-induced cytotoxicity. To investigate this possibility, HEK293-hOCT2 cells were first incubated with CDDP or OX (100 µM) under fed (DMEM) or starvation (EBSS, stv) conditions for 6 h. Then, incubation medium was removed, and cells were further incubated with DMEM or, only for CDDP experiments, with EBSS for 48 h. As shown in Figure 7, HEK293-hOCT2 cells were less sensitive to CDDP cytotoxicity when they were incubated under starvation, providing evidence that starvation offers protection against CDDP-associated cytotoxicity. Interestingly, performing postincubation under starvation conditions further reduced CDDP cellular toxicity. Conversely, starvation failed to protect against OX cytotoxicity and even increased its harmful effects. For this reason, further experiments where cells were starved after OX treatment were not performed.

**Figure 8:**
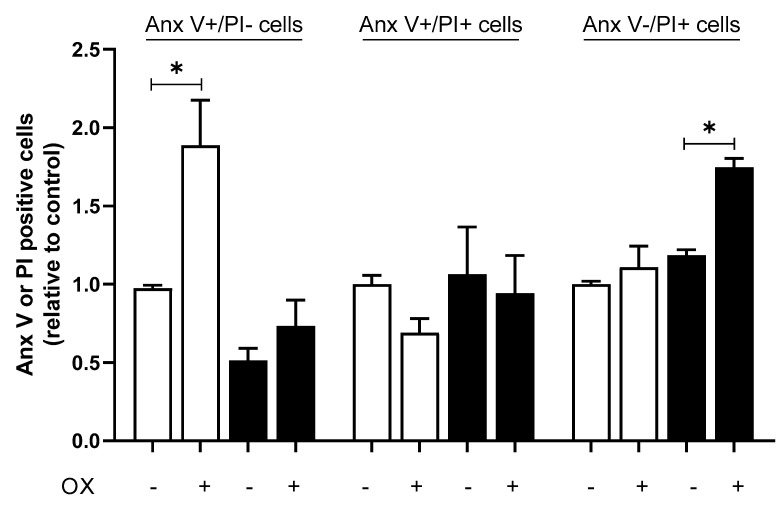
Cell death in HEK293-hOCT2 cells treated with OX under fed and starvation conditions. Apoptosis and necrosis were measured by means of annexin V (Anx V) staining and propidium iodide (PI) uptake in HEK293-hOCT2 cells after 24 h incubation with 100 µM OX under fed (open bars) or starvation (closed bars) conditions. Early apoptotic cells show Anx V positive (+)/PI negative (−) staining patterns; late apoptotic cells exhibited Anx V+/PI+ staining patterns due to a loss of plasma membrane integrity. Necrotic cells were defined as Anx V−/PI+ cells. Data are expressed relative to cells incubated with fed medium without OX and given as mean ± SEM of 3 independent experiments. * show statistically significant difference (*p* < 0.05) between the indicated groups (unpaired *t*-test).

Rather than contradictory, the finding regarding OX treatment just uncovers the fact that, in some circumstances, autophagy is not sufficient to mitigate cell damage or promote cell survival [35]. In this context, we analyzed the impact of OX on the levels of cell death by measuring apoptosis and necrosis in HEK293-hOCT2 cells. Cells treated 24 h with 100 µM OX under fed or starvation conditions were evaluated by means of Annexin V staining (Anx V, an apoptosis marker) and propidium iodide uptake (PI, a necrosis marker) by using flow cytometry analysis. As shown in Figure 8, OX significantly induced apoptosis (increased number of Anx V+/PI− cells) when incubation was performed under the fed condition only, while the treatment under starvation conditions predominantly caused cell necrosis (increased number of Anx V−/PI+ cells). Increased levels of cell death, more specifically increased necrotic rates, corroborate the higher degree of toxicity observed when OX was applied under starvation condition. In addition, it helps to explain the lack of protection expected for starvation under our conditions (Figure 7). A representative flow cytometry plot is given in Figure S1 of Supplementary Material.

## 3. Discussion

CDDP and OX are highly effective chemotherapeutic drugs, which are often used in the treatment of epithelial malignancies [4]. However, their use is limited by the occurrence of severe adverse events. Therefore, new strategies to minimize the adverse events are necessary to improve cancer chemotherapeutic treatment with these drugs. hOCT2 is a membrane transporter, which is involved in the development of unwanted side effects induced by Pt derivatives, but apparently does not play a role for their anticancer activity. Autophagy seems to be activated as a cellular protective mechanism following exposition to chemotherapeutic drugs. Therefore, it could be a promising target to reduce unwanted toxic effects of chemotherapy [36]. In this study, we analyzed whether (a) CDDP and OX induce autophagy; (b) hOCT2 is critical for this effect and (c) autophagy modulates CDDP and OX cellular toxicity.

Starvation is a well-known factor to induce autophagy in cells [37]. By depriving the cells of essential nutrients, the subsequent stress condition leads to an increased degradation of proteins and organelles. Interestingly, HEK293 cells show an increased modification of LC3 I to LC3 II after 6 h under starvation conditions (Figure 1A,B). The high expression of hOCT2 in HEK-hOCT2 cells seems to increase autophagic activity (Figure 1C,D), suggesting a higher translation and energy consumption in hOCT2 overexpressing cells as well as a growing rate of misfolded proteins to be degraded via the autophagosome [38]. Using this short incubation time (6 h), we decided to measure autophagy and autophagic flux using Western blot analysis of LC3 II/I expression ratio and p62 expression. Measuring protein expression is an important method for investigating the process of autophagy, especially when focusing on autophagic flux [39].

CDDP and OX are both substrates of hOCT2, which is believed to play a role in mediating severe nephro-, oto- and neuro-toxicity after chemotherapy [4]. The Pt agents are recognized inductors of autophagy, which works as a cytoprotective mechanism during the chemotherapeutic treatment [14,15,40]. Both CDDP and OX reduced p62 level in HEK293-hOCT2 cells under fed and starvation conditions (Figure 2), while in HEK293-WT cells, no or only minimal effects of CDDP/OX on autophagy activity were measured. Then, the autophagic flux was evaluated by treating cells with bafilomycin A1, which is known to inhibit the fusion of autophagosomes with lysosomes. Hereby we observed a significant increase in p62 expression level under bafilomycin A1 and OX treatment compared to a single OX treatment, indicating an inhibition of OX-induced autophagy (Figure 3).

To confirm this result, we used the conversion of LC3 I to LC3 II as an alternative autophagy marker (Figure 4). By measuring the autophagic flux under inhibition of autophagy with bafilomycin A1 again, we were able to confirm a significant increase in LC3 II in OX and bafilomycin A1 cotreated cells compared to cells treated only with OX.

Because autophagy is an important mechanism in stress conditions such as nutrient deprivation or ER stress, its activity is tightly regulated [41]. One of the key regulators of autophagy is mTORC1, which also regulates cell growth, cell proliferation, protein synthesis and transcription [42]. Upon amino acid deprivation, mTORC1 is inhibited, and autophagy is subsequently activated, indicating an inverse coupling mechanism [43]. One important downstream target of mTORC1 is p70 S6-kinase, which upon activation phosphorylates and activates the S6 protein [44]. By measuring the phosphorylated S6/S6 ratio, we could confirm that starvation induced autophagy (Figure 5), as shown by the decreased activity of the mTORC1 pathway. However, CDDP or OX incubation did not change the activity of the mTORC1 pathway. It is well-known that several mTOR-independent autophagy pathways exist (such as Ca^2+^, AMPK, MAPK/JNK, reactive oxygen species (ROS), HIF-1*α*; for a review see [45]), and therefore, it can be speculated that in this setting, CDDP- and OX-associated autophagy is mediated by a noncanonical mTOR-independent signaling pathway.

Autophagy is usually thought to be a cell-survival mechanism, that, for example, prevents the accumulation of toxic cellular waste products and generates energy and macromolecular precursors [46]. By inhibiting this process with inhibitors such as bafilomycin A1, nuclear fragmentation, apoptosis and oxidative stress in cisplatin-treated cells are enhanced [47]. The inhibition of autophagy with bafilomycin A1 and a simultaneous CDDP or OX incubation showed in HEK293-WT and HEK293-hOCT2 cells a greater toxic effect than the treatment with the Pt derivatives alone (Figure 6). This indicates in both WT and hOCT2-overexpressing cells the important cytoprotective role of CDDP/OX-induced autophagy. To confirm this cytoprotective function of autophagy, we increased autophagy activity by starving the cells during or after the CDDP/OX treatment (Figure 7). By measuring the cell viability in HEK293-hOCT2 cells, we observed a stronger protective effect against CDDP-mediated toxicity by prolonging starvation during a 48 h postincubation period after CDDP treatment. Longer starvation postincubation time (72 h) reverted the protective effect to a more toxic one (not shown), probably because the damage induced by starvation became prevalent. For OX, a different behavior was observed: starvation during OX incubation period increased cellular OX toxicity (Figure 7). This may be explained by the observation that even though starvation protects against OX-induced apoptosis, it increased necrotic cell death (Figure 8).

It is important to note that, although autophagy is considered a cytoprotective process, in certain circumstances it fails to prevent cell damage and can even induce cell death directly and indirectly, taking part in a complex cross talk with apoptosis and necrosis [35,48]. For example, starvation is known to induce expression of the cyclin-dependent kinase inhibitor p21 (WAF1/Cip1), at least in the human osteoblastic cell line MG63 [49]. Interestingly, in a toxin-induced model of liver injury, p21 is required for necrosis, but it inhibits apoptosis [50]. Therefore, it can be speculated that the above-described experimental starvation induces p21 expression, which in turn decreases OX-induced apoptosis and causes cell necrosis.

To improve the results of chemotherapeutic therapy with platinum agents in the treatment of solid cancer, the control of side effects is essential. It has been demonstrated in mice that caloric restriction is effective in ameliorating cisplatin-induced nephrotoxicity presumably by stimulation of autophagy [51]. According to our data, autophagy induction by starvation can be effective in protecting hOCT2-expressing cells against CDDP (however only under very narrow experimental conditions) but not OX toxic effects. As a possible clinically feasible approach to stimulate autophagy, caloric restriction or mTORC inhibitors such as everolimus or sirolimus could be used to imitate starvation-induced autophagy [52]. In this work, we found no evidence that CDDP and OX influence mTORC activity, and therefore, mTORC inhibition can be a useful approach to modulate autophagic activity independently from CDDP and OX effects. Moreover, several studies have demonstrated a beneficial effect by combining chemotherapeutic drugs with an mTORC inhibitor to improve the antitumoral efficacy of chemotherapy [52,53,54].

Autophagy is highly discussed in cancer therapy as it plays a dual role as a tumor suppressor and a tumor promotor factor [55]. Autophagy is responsible for degrading damaged proteins and toxic waste products, reducing reactive oxygen species and DNA damage, and therefore protects the cells from developing cancer [56]. On the other hand, by securing energy and macromolecular precursors for the rapidly growing cancer cells, it enhances tumor growth and cell survival [56]. Additionally, cancer cells treated with CDDP develop chemoresistance, which is probably linked to an increased autophagy level [57,58]. Moreover, we have shown that induction of autophagy by starvation can protect the cells only in a close range of conditions, and therefore, establishment of a clinically useful therapeutic protocol using autophagy activation for protection against side-effects of cancer chemotherapy with platinum derivatives is probably very difficult.

In conclusion, our present study showed that CDDP and OX are capable of inducing autophagy mainly in HEK293-hOCT2 cells. Our results also demonstrated that inhibition of autophagy increases cellular death associated with platinum agents. Therefore, activation of autophagy could be a potential protective strategy against CDDP- and OX-induced cytotoxicity, especially in cells and tissues expressing hOCT2.

## 4. Materials and Methods

### 4.1. Cell Culture

HEK293-WT and HEK293 stably overexpressing hOCT2 were a generous gift of Prof. Koepsell [8,59]. Cells were cultivated at 37 °C and 5% CO_2_ in DMEM low glucose (1 g/L, Sigma-Aldrich, Munich, Germany) with 10% FCS (Sigma-Aldrich), 1% penicillin/streptomycin (Merck, Darmstadt, Germany) and additional 50 mg/mL G418 (PAN-Biotech, Aidenbach, Germany) as a selection antibiotic for HEK293-hOCT2 cells. Cells were seeded in 12-well plates (Greiner, Frickenhausen, Germany) with DMEM low glucose. At 90% confluency, cells were washed with phosphate-buffered saline (PBS, Sigma-Aldrich). To study the effects of autophagy induction, cells were incubated with DMEM low glucose (fed condition) or Earle’s Balanced Salt Solution (EBSS, Sigma-Aldrich, starvation condition) for 2, 4, or 6 h. Since 6 h starvation clearly activated autophagy (Figure 1), further experiments were performed after 6 h incubation with 100 µM CDDP or OX (Teva Pharm, Ulm, Germany) under fed and starvation conditions, as described above, in the presence or absence of 100 nM bafilomycin A1 (InvivoGen Europe, Toulouse, France) as an inhibitor of lysosomal acidification and therefore of autophagy. The concentration of platinum derivatives used in the present work reflects what used in other in vitro studies [60,61]; moreover, in vitro studies show that at this concentration, these substances show a robust interaction with hOCT2 [8,9].

### 4.2. Western Blot Analysis of CDDP and OX Effects on Autophagy

At the end of incubation period, cells were lysed using an appropriate volume of Laemmli buffer (120 mM Tris-HCl pH 6.8, 4% sodium dodecyl sulfate (SDS), 20% glycerol, 0.02% bromphenol blue) supplemented with 30 µL of 1 M DTT/mL buffer. Cells were scratched off the plate into a reaction tube, which was boiled at 95 °C for 5 min. Samples were then placed in an ultrasonic bath for 10 min and centrifuged at 13,800× *g* for 5 min at 4 °C. Proteins were separated using SDS polyacrylamide gel electrophoresis (SDS-PAGE) (BioRad, Munich, Germany). After protein separation, samples were then transferred to a nitrocellulose membrane. Upon completion of protein transfer, unspecific binding to the membrane was blocked by 1 h of incubation with 5% bovine serum albumin (BSA) dissolved in tris-buffered saline with Tween 20 (TBS-T). Then, the membrane was incubated with primary antibodies at 4 °C overnight. The antibodies were diluted in TBS-T with 5% BSA as follows: *α*-actinin (Cell Signaling, Danvers, USA) 1:1000; p62-lck (BD Biosciences, San Jose, USA) 1:1000; LC3B (Novus Biologicals, Littleton, USA) 1:4000; S6 (Cell Signaling) 1:1000; pS6 (Cell Signaling) 1:1000; hOCT2 (a generous gift by Prof. Koepsell, Würzburg University [62]) 1:500; GAPDH (Cell Signaling) 1:1000. After this, the membrane was washed three times with TBS-T and incubated with horseradish peroxidase-coupled secondary antibodies (*α*-rabbit/*α*-mouse, Dako, Glostrup, Denmark, 1:10,000) for 1 h. After additional TBS-T washing steps, the signals were detected using a chemiluminescence detection reagent (Clarity, BioRad) and an Azure biosystems imager (c600, Azure Biosystems, Dublin, USA). Band intensity was quantified by densitometry using the software ImageJ [63].

### 4.3. Cell Viability

To determine the effects of autophagy inhibition on CDDP-associated cellular toxicity, the 3-(4,5-dimethylthiazol-2-yl)-2,5-diphenyltetrazoliumbromid (MTT) test was used. Briefly, HEK293-WT and HEK293-hOCT2 cells were seeded in a 96-well plate and cultivated at 37 °C with 5% CO_2_. At 80–90% confluency, the cells were incubated with DMEM low glucose or EBSS containing 100 µM CDDP or OX with or without 100 nM bafilomycin A1 for 6 h. Subsequently, cells were postincubated with drug-free DMEM low glucose, or in some cases (when applying CDDP treatment) with EBSS for 48 h. At the end of the postincubation time, 10 µL MTT reagent (Sigma-Aldrich, 5 mg/mL MTT in PBS) were added, and the cells were incubated for a further 3 h. To stop the reaction, 100 µL of lysis solution (10% SDS, 42% dimethylformamide, 1% HCl 1 M) was added to each well and the cells were incubated at room temperature overnight. Absorbance was measured spectrophotometrically with a microplate reader (Infinite M200, Tecan group, Männedorf, Swizerland) at 590 nm. Background absorbance was calculated by measuring wells with medium but without cells.

### 4.4. Flow Cytometry

Apoptosis was determined based on the interaction between annexin V and externalized phosphatidylserine using fluorescence-activated cell sorting (FACS), as described previously [8]. Evaluation of cell necrosis was performed by propidium iodide uptake by nonpermeabilized cells. HEK293-hOCT2 cells were seeded in a 6-well plate and cultivated at 37 °C with 5% CO_2_. At 80–90% confluency, the cells were incubated for 24 h with DMEM low glucose or EBSS containing 100 µM OX. At the end of incubation time, cells were resuspended and washed in 500 µL FACS-Buffer (PBS with Ca^2+^ and Mg^2+^ containing 0.5% fetal calf serum and 0.5% NaN_3_), and incubated with 5 µL annexin V-APC and propidium iodide (5 μg/mL) (BD Biosciences, San Jose, CA, USA) in 100 µL FACS-Buffer for 25 min at 4 °C. Cells were washed again, resuspended in 500 µL of FACS-Buffer and immediately analyzed on a FACSCalibur flow cytometer (BD Biosciences, San Jose, CA, USA). At least 20,000 events were triggered by the forward-scatter and side-scatter light, and samples were analyzed by using FlowJo_v10.6.2 software (BD Biosciences). Cells stained positive for annexin V-APC (FL-4) and negative for propidium iodide (FL-2) were considered as early apoptotic, whereas double-positive cells were defined as late apoptotic. Cells stained positive for propidium iodide only were primarily considered necrotic.

### 4.5. Statistical Analysis

Data are presented as mean ± SEM of at least three independent measurements. Statistical analyses were performed with GraphPad Prism software 5.0 (GraphPad Software, Inc., San Diego, CA, USA) using one way ANOVA with Tukey’s multiple comparison test and paired/unpaired *t*-test as appropriate.

## Data Availability

Data is contained within the article and Supplementary Materials.

## Supplementary Materials

The following supporting information can be downloaded at: www.mdpi.com/article/10.3390/ijms23031090/s1.

## References

[1] Wang D., Lippard S.J. (2005). Cellular Processing of Platinum Anticancer Drugs. Nat. Rev. Drug Discov..

[2] Ciarimboli G. (2011). Role of Organic Cation Transporters in Drug-Induced Toxicity. Expert Opin. Drug Metab. Toxicol..

[3] Yimit A., Adebali O., Sancar A., Jiang Y. (2019). Differential Damage and Repair of DNA-Adducts Induced by Anti-Cancer Drug Cisplatin across Mouse Organs. Nat. Commun..

[4] Harrach S., Ciarimboli G. (2015). Role of Transporters in the Distribution of Platinum-Based Drugs. Front. Pharm..

[5] Fuertes M., Castilla J., Alonso C., Perez J. (2002). Novel Concepts in the Development of Platinum Antitumor Drugs. Curr. Med. Chem. Anti Cancer Agents.

[6] Alian O.M., Azmi A.S., Mohammad R.M. (2012). Network Insights on Oxaliplatin Anti-cancer Mechanisms. Clin. Transl. Med..

[7] de Luca A., Parker L.J., Ang W.H., Rodolfo C., Gabbarini V., Hancock N.C., Palone F., Mazzetti A.P., Menin L., Morton C.J. (2019). A Structure-Based Mechanism of Cisplatin Resistance Mediated by Glutathione Transferase P1-1. Proc. Natl. Acad. Sci. USA.

[8] Ciarimboli G., Ludwig T., Lang D., Pavenstädt H., Koepsell H., Piechota H.J., Haier J., Jaehde U., Zisowsky J., Schlatter E. (2005). Cisplatin Nephrotoxicity Is Critically Mediated via the Human Organic Cation Transporter 2. Am. J. Pathol..

[9] Sprowl J.A., Ciarimboli G., Lancaster C.S., Giovinazzo H., Gibson A.A., Du G., Janke L.J., Cavaletti G., Shields A.F., Sparreboom A. (2013). Oxaliplatin-Induced Neurotoxicity Is Dependent on the Organic Cation Transporter OCT2. Proc. Natl. Acad. Sci. USA.

[10] Yonezawa A., Masuda S., Yokoo S., Katsura T., Inui K.-I. (2006). Cisplatin and Oxaliplatin, but Not Carboplatin and Nedaplatin, Are Substrates for Human Organic Cation Transporters (SLC22A1-3 and Multidrug and Toxin Extrusion Family). J. Pharmacol. Exp. Ther..

[11] Huang K.M., Leblanc A.F., Uddin M.E., Kim J.Y., Chen M., Eisenmann E.D., Gibson A.A., Li Y., Hong K.W., DiGiacomo D. (2020). Neuronal Uptake Transporters Contribute to Oxaliplatin Neurotoxicity in Mice. J. Clin. Investig..

[12] Ciarimboli G., Deuster D., Knief A., Sperling M., Holtkamp M., Edemir B., Pavenstädt H., Lanvers-Kaminsky C., Am Zehnhoff-Dinnesen A., Schinkel A.H. (2010). Organic Cation Transporter 2 Mediates Cisplatin-Induced Oto- and Nephrotoxicity and Is a Target for Protective Interventions. Am. J. Pathol..

[13] Sprowl J.A., van Doorn L., Hu S., van Gerven L., de Bruijn P., Li L., Gibson A.A., Mathijssen R.H., Sparreboom A. (2013). Conjunctive Therapy of Cisplatin with the OCT2 Inhibitor Cimetidine: Influence on Antitumor Efficacy and Systemic Clearance. Clin. Pharmacol. Ther..

[14] Yang C., Kaushal V., Shah S.V., Kaushal G.P. (2008). Autophagy Is Associated with Apoptosis in Cisplatin Injury to Renal Tubular Epithelial Cells. Am. J. Physiol. Ren. Physiol..

[15] Shi Y., Tang B., Yu P.-W., Tang B., Hao Y.-X., Lei X., Luo H.-X., Zeng D.-Z. (2012). Autophagy Protects against Oxaliplatin-Induced Cell Death via ER Stress and ROS in Caco-2 Cells. PLoS ONE.

[16] Fung C., Lock R., Gao S., Salas E., Debnath J. (2008). Induction of Autophagy during Extracellular Matrix Detachment Promotes Cell Survival. Mol. Biol. Cell.

[17] Shi F., Luo D., Zhou X., Sun Q., Shen P., Wang S. (2021). Combined Effects of Hyperthermia and Chemotherapy on the Regulate Autophagy of Oral Squamous Cell Carcinoma Cells under a Hypoxic Microenvironment. Cell Death Discov..

[18] Yang H.-Z., Ma Y., Zhou Y., Xu L.-M., Chen X.-J., Ding W.-B., Zou H.-B. (2015). Autophagy Contributes to the Enrichment and Survival of Colorectal Cancer Stem Cells under Oxaliplatin Treatment. Cancer Lett..

[19] Qiang L., Wu C., Ming M., Viollet B., He Y.-Y. (2013). Autophagy Controls P38 Activation to Promote Cell Survival under Genotoxic Stress. J. Biol. Chem..

[20] Nishida K., Kyoi S., Yamaguchi O., Sadoshima J., Otsu K. (2009). The Role of Autophagy in the Heart. Cell Death Differ..

[21] Luo L., Zhang P., Zhu R., Fu J., Su J., Zheng J., Wang Z., Wang D., Gong Q. (2017). Autophagy Is Rapidly Induced by Salt Stress and Is Required for Salt Tolerance in Arabidopsis. Front. Plant Sci..

[22] Xiong Y., Contento A.L., Bassham D.C. (2005). AtATG18a Is Required for the Formation of Autophagosomes during Nutrient Stress and Senescence in Ara-bidopsis Thaliana. Plant J..

[23] He C., Klionsky D.J. (2009). Regulation Mechanisms and Signaling Pathways of Autophagy. Annu. Rev. Genet..

[24] Mizushima N., Yoshimori T. (2007). How to Interpret LC3 Immunoblotting. Autophagy.

[25] Buccarelli M., Marconi M., Pacioni S., de Pascalis I., D’Alessandris Q.G., Martini M., Ascione B., Malorni W., Larocca L.M., Pallini R. (2018). Inhibition of Autophagy Increases Susceptibility of Glioblastoma Stem Cells to Temozolomide by Igniting Ferroptosis. Cell Death Dis..

[26] Xu L., Qu X.-J., Liu Y.-P., Xu Y.-Y., Liu J., Hou K.-Z., Zhang Y. (2011). Protective Autophagy Antagonizes Oxaliplatin-Induced Apoptosis in Gastric Cancer Cells. Chin. J. Cancer.

[27] Huang R., Xu Y., Wan W., Shou X., Qian J., You Z., Liu B., Chang C., Zhou T., Lippincott-Schwartz J. (2015). Deacetylation of Nuclear LC3 Drives Autophagy Initiation under Starvation. Mol. Cell.

[28] Scott R.C., Schuldiner O., Neufeld T.P. (2004). Role and Regulation of Starvation-Induced Autophagy in the Drosophila Fat Body. Dev. Cell.

[29] Liu W.J., Ye L., Huang W.F., Guo L.J., Xu Z.G., Wu H.L., Yang C., Liu H.F. (2016). p62 Links the Autophagy Pathway and the Ubiqutin–Proteasome System upon Ubiquitinated Protein Degradation. Cell. Mol. Biol. Lett..

[30] Yuan N., Song L., Zhang S., Lin W., Cao Y., Xu F., Fang Y., Wang Z., Zhang H., Li X. (2015). Bafilomycin A1 Targets Both Autophagy and Apoptosis Pathways in Pediatric B-Cell Acute Lymphoblastic Leukemia. Haematologica.

[31] Tanida I., Minematsu-Ikeguchi N., Ueno T., Kominami E. (2005). Lysosomal Turnover, but Not a Cellular Level, of Endogenous LC3 Is a Marker for Autophagy. Autophagy.

[32] Du W., Gerald D., Perruzzi C.A., Rodriguez-Waitkus P., Enayati L., Krishnan B., Edmonds J., Hochman M.L., Lev D.C., Phung T.L. (2013). Vascular Tumors Have Increased P70 S6-Kinase Activation and Are Inhibited by Topical Rapamycin. Lab. Investig..

[33] Zhang D., Pan J., Xiang X., Liu Y., Dong G., Livingston M.J., Chen J.-K., Yin X.-M., Dong Z. (2017). Protein Kinase C δ Suppresses Autophagy to Induce Kidney Cell Apoptosis in Cisplatin Nephrotoxicity. J. Am. Soc. Nephrol..

[34] Zhang S., Lovejoy K.S., Shima J.E., Lagpacan L.L., Shu Y., Lapuk A., Chen Y., Komori T., Gray J.W., Chen X. (2006). Organic Cation Transporters Are Determinants of Oxaliplatin Cytotoxicity. Cancer Res..

[35] Noguchi M., Hirata N., Tanaka T., Suizu F., Nakajima H., Chiorini J.A. (2020). Autophagy as a Modulator of Cell Death Machinery. Cell Death Dis..

[36] Levy J.M.M., Towers C.G., Thorburn A. (2017). Targeting Autophagy in Cancer. Nat. Rev. Cancer.

[37] Ravanan P., Srikumar I.F., Talwar P. (2017). Autophagy: The Spotlight for Cellular Stress Responses. Life Sci..

[38] Moriya H. (2015). Quantitative Nature of Overexpression Experiments. Mol. Biol. Cell.

[39] Yoshii S.R., Mizushima N. (2017). Monitoring and Measuring Autophagy. Int. J. Mol. Sci..

[40] Periyasamy-Thandavan S., Jiang M., Wei Q., Smith R., Yin X.-M., Dong Z. (2008). Autophagy Is Cytoprotective during Cisplatin Injury of Renal Proximal Tubular Cells. Kidney Int..

[41] Rabanal-Ruiz Y., Otten E.G., Korolchuk V.I. (2017). MTORC1 as the Main Gateway to Autophagy. Essays Biochem..

[42] Szwed A., Kim E., Jacinto E. (2021). Regulation and Metabolic Functions of MTORC1 and MTORC2. Physiol. Rev..

[43] Kim Y.C., Guan K.-L. (2015). MTOR: A Pharmacologic Target for Autophagy Regulation. J. Clin. Investig..

[44] Dufner A., Thomas G. (1999). Ribosomal S6 Kinase Signaling and the Control of Translation. Exp. Cell Res..

[45] Al-Bari M.A.A., Xu P. (2020). Molecular Regulation of Autophagy Machinery by MTOR-dependent and -independent Pathways. Ann. N. Y. Acad. Sci..

[46] Rabinowitz J.D., White E. (2010). Autophagy and Metabolism. Science.

[47] Lin J.-F., Lin Y.-C., Tsai T.-F., Chen H.-E., Chou K.-Y., Hwang I.-S. (2017). Cisplatin Induces Protective Autophagy through Activation of BECN1 in Human Bladder Cancer Cells. Drug Des. Dev. Ther..

[48] Bialik S., Dasari S.K., Kimchi A. (2018). Autophagy-Dependent Cell Death—Where, How and Why a Cell Eats Itself to Death. J. Cell Sci..

[49] Funato N., Ohtani K., Ohyama K., Kuroda T., Nakamura M. (2001). Common Regulation of Growth Arrest and Differentiation of Osteoblasts by Helix-Loop-Helix Factors. Mol. Cell. Biol..

[50] Kwon Y.H., Jovanovic A., Serfas M.S., Tyner A.L. (2003). The Cdk Inhibitor p21 Is Required for Necrosis, but It Inhibits Apoptosis Following Toxin-Induced Liver Injury. J. Biol. Chem..

[51] Späth M.R., Bartram M.P., Palacio-Escat N., Hoyer K.J.R., Debes C., Demir F., Schroeter C.B., Mandel A.M., Grundmann F., Ciarimboli G. (2019). The Proteome Microenvironment Determines the Protective Effect of Preconditioning in Cisplatin-Induced Acute Kidney Injury. Kidney Int..

[52] Kopeina G.S., Senichkin V.V., Zhivotovsky B. (2017). Caloric Restriction—A Promising Anti-Cancer Approach: From Molecular Mechanisms to Clinical Trials. Biochim. Biophys. Acta Rev. Cancer.

[53] Eroglu Z., Tawbi H.A., Hu J., Guan M., Frankel P.H., Ruel N.H., Wilczynski S., Christensen S., Gandara D.R., Chow W.A. (2015). A Randomised Phase II Trial of Selumetinib vs Selumetinib plus Temsirolimus for Soft-Tissue Sarcomas. Br. J. Cancer.

[54] Hess G., Keller U., Scholz C.W., Witzens-Harig M., Atta J., Buske C., Kirschey S., Ruckes C., Medler C., van Oordt C. (2015). Safety and Efficacy of Temsirolimus in Combination with Bendamustine and Rituximab in Relapsed Mantle Cell and Follicular Lymphoma. Leukemia.

[55] Yun C., Lee S. (2018). The Roles of Autophagy in Cancer. Int. J. Mol. Sci..

[56] White E. (2015). The Role for Autophagy in Cancer. J. Clin. Investig..

[57] Xia M., Yu H., Gu S., Xu Y., Su J., Li H., Kang J., Cui M. (2014). P62/SQSTM1 Is In-volved in Cisplatin Resistance in Human Ovarian Cancer Cells via the Keap1-Nrf2-ARE System. Int. J. Oncol..

[58] Ren J.-H., He W.-S., Nong L., Zhu Q.-Y., Hu K., Zhang R.-G., Huang L.-L., Zhu F., Wu G. (2010). Acquired Cisplatin Resistance in Human Lung Adenocarcinoma Cells Is Associated with Enhanced Autophagy. Cancer Biother. Radio-Pharm..

[59] Lee W.-K., Reichold M., Edemir B., Ciarimboli G., Warth R., Koepsell H., Thévenod F. (2009). Organic Cation Transporters OCT1, 2, and 3 Mediate High-Affinity Transport of the Mutagenic Vital Dye Ethidium in the Kidney Proximal Tubule. Am. J. Physiol. Ren. Physiol..

[60] Legin A.A., Schintlmeister A., Sommerfeld N.S., Eckhard M., Theiner S., Reipert S., Strohhofer D., Jakupec M.A., Galanski M., Wagner M. (2021). Nano-Scale Imaging of Dual Stable Isotope Labeled Oxaliplatin in Human Colon Cancer Cells Reveals the Nucleolus as a Putative Node for Therapeutic Effect. Nanoscale Adv..

[61] Ludwig T., Riethmüller C., Gekle M., Schwerdt G., Oberleithner H. (2004). Nephrotoxicity of Platinum Complexes Is Related to Basolateral Organic Cation Transport. Kidney Int..

[62] Biermann J., Lang D., Gorboulev V., Koepsell H., Sindic A., Schröter R., Zvirbliene A., Pavenstädt H., Schlatter E., Ciarimboli G. (2006). Characterization of Regulatory Mechanisms and States of Human Organic Cation Transporter 2. Am. J. Physiol. Cell Physiol..

[63] Schneider C.A., Rasband W.S., Eliceiri K.W. (2012). NIH Image to ImageJ: 25 Years of Image Analysis. Nat. Methods.

