# Supplementary material for: Role of Organic Cation Transporter 2 in Autophagy Induced by Platinum Derivatives
Source: Int J Mol Sci. 2022 Jan 19;23(3):1090. doi: 10.3390/ijms23031090 (PMC8834759; doi:10.3390/ijms23031090)
Supplement: Supplementary file 1 [file ijms-23-01090-s001.zip › ijms-1535748-supplementary.pdf]

Supplementary Material

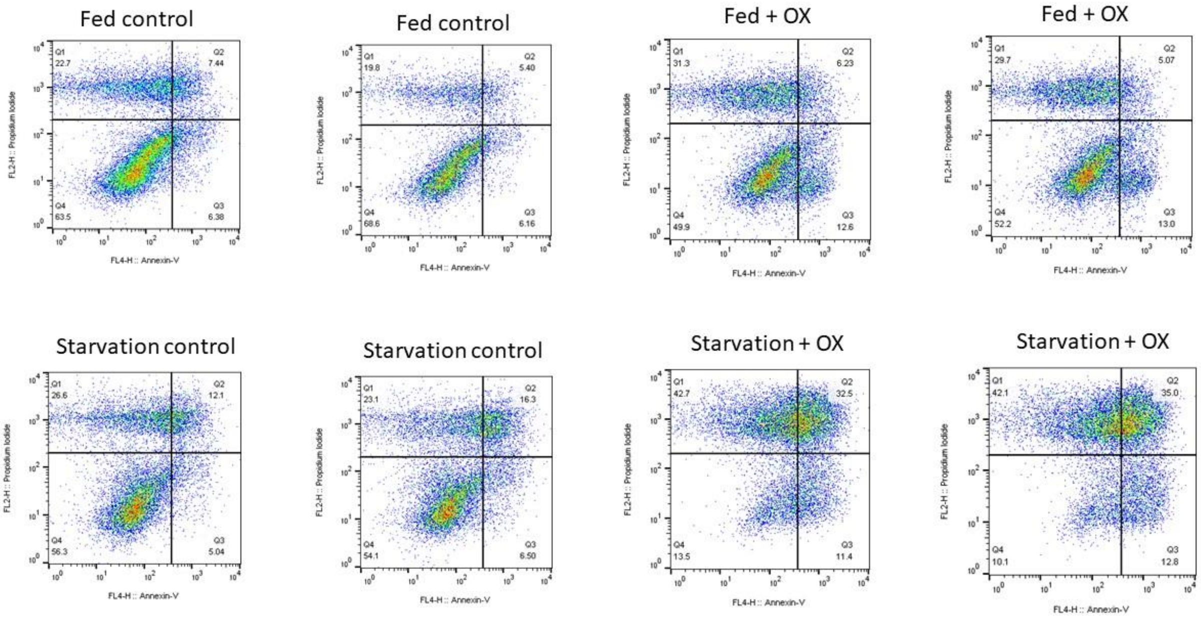

Figure S1. This figure shows an example of fluorescence-activated cell sorting measurement of annexin V (apoptosis marker, x-axis) and propidium iodide (necrosis marker, y-axis) performed using HEK293-hOCT2 cells after 24 h treatment with 100 μM OX under fed or starvation conditions. Control experiments were performed under fed or starvation conditions but without OX treatment. Values in the different quadrants (Q) show the percentage of cells in the quadrant referred to total cell population.

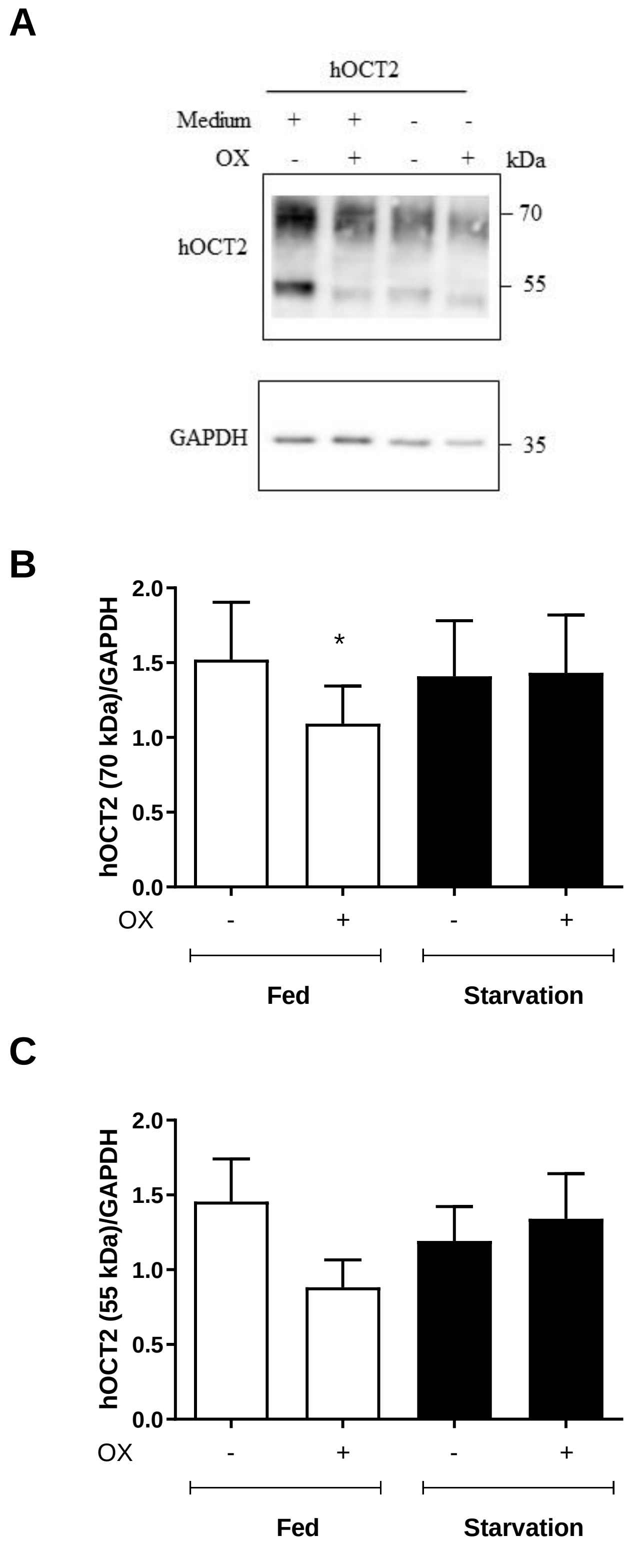

Figure S2. Protein hOCT2/GAPDH expression ratio in dependence from starvation and/or OX treatment measured by Western blot analysis in HEK293-hOCT2 cells. Cells were incubated for 6 h with DMEM (fed, open bars)- or EBSS (starvation, closed bars)- medium in the presence or not of 100 μM OX. GAPDH served as a loading control. Panel A shows an example of a Western blot experiment: since the antibody used here for hOCT2 labelling [1] is known to specifically detect the glycosylated and non-glycosylated form of hOCT2, with an apparent molecular weight of 70 and 55 kDa, respectively [2], the two form of hOCT2 could be evaluated separately. Panel A und panel B shows the quantification of the

results obtained from 4 independent experiments (mean ±SEM). Panel A shows the glycosylated hOCT2/GAPDH and panel B the non-glycosylated hOCT2/GAPDH expression ratios. The star shows a statistically significant difference between the indicated group and the one without oxaliplatin treatment ($p < 0.05$, unpaired t-test).

1. Biermann, J.; Lang, D.; Gorboulev, V.; Koepsell, H.; Sindic, A.; Schröter, R.; Zvirbliene, A.; Pavenstädt, H.; Schlatter, E.; Ciarimboli, G. Characterization of Regulatory Mechanisms and States of Human Organic Cation Transporter 2. *Am.J.Physiol.Cell.Physiol.* **2006**, *290*, C1521–C1531.

2. Brast, S.; Grabner, A.; Sucic, S.; Sitte, H.H.; Hermann, E.; Pavenstädt, H.; Schlatter, E.; Ciarimboli, G. The Cysteines of the Extracellular Loop Are Crucial for Trafficking of Human Organic Cation Transporter 2 to the Plasma Membrane and Are Involved in Oligomerization. *FASEB journal : official publication of the Federation of American Societies for Experimental Biology* **2012**, *26*, 976–986, doi:10.1096/fj.11-180679.
